# The yeast molecular chaperone, Hsp104, influences transthyretin aggregate formation

**DOI:** 10.3389/fnmol.2022.1050472

**Published:** 2022-12-16

**Authors:** Adam S. Knier, Emily E. Davis, Hannah E. Buchholz, Jane E. Dorweiler, Lauryn E. Flannagan, Anita L. Manogaran

**Affiliations:** Department of Biological Sciences, Marquette University, Milwaukee, WI, United States

**Keywords:** Hsp104, transthyretin, molecular chaperone, protein aggregation, transthyretin amyloidosis, amyloid, polyneuropathy, aging

## Abstract

Patients with the fatal disorder Transthyretin Amyloidosis (ATTR) experience polyneuropathy through the progressive destruction of peripheral nervous tissue. In these patients, the transthyretin (TTR) protein dissociates from its functional tetrameric structure, misfolds, and aggregates into extracellular amyloid deposits that are associated with disease progression. These aggregates form large fibrillar structures as well as shorter oligomeric aggregates that are suspected to be cytotoxic. Several studies have shown that these extracellular TTR aggregates enter the cell and accumulate intracellularly, which is associated with increased proteostasis response. However, there are limited experimental models to study how proteostasis influences internalized TTR aggregates. Here, we use a humanized yeast system to recapitulate intracellular TTR aggregating protein *in vivo*. The yeast molecular chaperone Hsp104 is a disaggregase that has been shown to fragment amyloidogenic aggregates associated with certain yeast prions and reduce protein aggregation associated with human neurogenerative diseases. In yeast, we found that TTR forms both SDS-resistant oligomers and SDS-sensitive large molecular weight complexes. In actively dividing cultures, Hsp104 has no impact on oligomeric or large aggregate populations, yet overexpression of Hsp104 is loosely associated with an increase in overall aggregate size. Interestingly, a potentiating mutation in the middle domain of Hsp104 consistently results in an increase in overall TTR aggregate size. These data suggest a novel approach to aggregate management, where the Hsp104 variant shifts aggregate populations away from toxic oligomeric species to more inert larger aggregates. In aged cultures Hsp104 overexpression has no impact on TTR aggregation profiles suggesting that these chaperone approaches to shift aggregate populations are not effective with age, possibly due to proteostasis decline.

## Introduction

Onset of the progressively fatal disease Transthyretin Amyloidosis (ATTR) is associated with the amyloidogenic aggregation of the human transthyretin (TTR) protein. As a functional tetramer, TTR shuttles hormone and proteins within the bloodstream and CNS. However, the tetramer can dissociate with age, leading to the misfolding and subsequent aggregation of the TTR monomer. It is the tetramer dissociation that is considered the rate limiting step of the aggregation process (Hammarstrom et al., 2003). The misfolded monomer can assemble into small oligomeric species or large amyloid. *In vitro* evidence suggests that these small oligomeric TTR aggregates are cytotoxic (Dasari et al., 2019). Over time, the circulating TTR aggregates are deposited into the heart and peripheral nerves, leading to system organ failure and death (Gertz et al., 2015). Interestingly, approaches that shift the equilibrium away from cytotoxic oligomeric species and towards either monomeric protein or large amyloid may be a means to delay the disease.

Transthyretin aggregates have been observed within cells, detected as intracellular cytoplasmic deposits (Brett et al., 1999; Misumi et al., 2013). Cells with internalized TTR also display an upregulation of the HSF1 protein, a master regulator for heat shock related chaperones (Santos et al., 2010). Furthermore, the levels of several molecular chaperones appear to be increased in ATTR patients (Misumi et al., 2013; da Costa et al., 2015) suggesting a correlation between internalization and an *in vivo* molecular chaperone response.

Molecular chaperones play an important role in maintaining protein homeostasis by facilitating proper polypeptide folding while managing the accumulation of excess aberrantly folded proteins (Mogk et al., 2018). Elevated chaperone activity not only is associated with ATTR, but chaperones have been shown to contribute to the prevention of neurodegenerative disease onset, such as Alzheimer’s and Prion disease (Wentink et al., 2019; Tittelmeier et al., 2020). However, as cells age, the ability for chaperones to maintain intracellular proteostasis declines, resulting in protein aggregation and associated disease onset (Klaips et al., 2018).

The study of protein aggregation and chaperones in yeast has greatly advanced our mechanistic understanding of how chaperones influence protein aggregates in humans (Surguchov, 2021). The AAA+ hexameric ATPase, Hsp104, interacts with highly conserved Hsp70 and Hsp40 co-chaperones to fragment several yeast prions that are amyloidogenic. The Hsp104/70/40 complex extracts monomeric subunits from the middle of prion fibrils, thus fragmenting the aggregate into smaller pieces (Parsell et al., 1994; Glover and Lindquist, 1998; Shorter and Lindquist, 2004; Satpute-Krishnan et al., 2007; DeSantis et al., 2012). While Hsp104 is not found in metazoans, *in vitro* studies show that wildtype-Hsp104 can disassemble human amyloidogenic aggregating proteins (Lo Bianco et al., 2008; Liu et al., 2011; DeSantis et al., 2012; March et al., 2019). In contrast, *in vivo* studies indicate that wildtype-Hsp104 has little effect on aggregate disassembly, while mutations within the regulatory middle-domain can potentiate Hsp104 function resulting in aggregate disaggregation and suppression of cellular toxicity (Jackrel et al., 2014; March et al., 2020).

Several labs have used a yeast-based system in which intracellular TTR is expressed as a monomer, thus bypassing the rate limiting step of conversion from tetrameric to monomeric form, which allows for rapid TTR aggregation (Derkatch et al., 2004; Gomes et al., 2012; Verma et al., 2018). The aggregated TTR displays hallmarks of amyloidogenic protein including detection of SDS-resistant complexes (Gomes et al., 2012) and fluorescent puncta when fused to GFP (Derkatch et al., 2004; Verma et al., 2018). Here, we use this yeast-based system to explore how Hsp104 influences the formation of TTR aggregates *in vivo*. We find that TTR forms both SDS-resistant oligomers and aggregates of larger sizes. However, it appears that these SDS-resistant species also make up larger aggregates held together by SDS-sensitive bonds. While loss of Hsp104 reduces the abundance of detergent resistant TTR populations, it does not impact the size of SDS-resistant oligomers. While overexpression of wildtype Hsp104 is loosely associated with an increase in overall TTR aggregate size, a Hsp104 middle domain mutant containing a A503S substitution is associated with a more definitive increase in aggregate size. We suspect that this mutation plays an important role in shifting the equilibrium away from the oligomeric species towards larger aggregates. However, any Hsp104 specific influence observed on TTR aggregates during logarithmic growth no longer occurs in saturated cultures, suggesting that Hsp104 alone cannot overcome age-related proteostasis decline.

## Materials and methods

### Strains, plasmids, and cultivation procedures

All yeast strains are derivatives of the 74-D694 background and listed in Supplementary Table 1. *hsp104Δ* 74-D694 strains were generated by standard homologous recombination as described in Manogaran et al. (2011). Primers to generate this strain (M515) are listed in Supplementary Table 2. Plasmids used in this study are listed in Supplementary Table 3. Note that centromeric plasmids are usually held at 2–10 copies per cell and 2 micron plasmids are held at 20–50 copies per cell (Karim et al., 2013). *hsp104Δ* strains transformed with the TTR-GFP plasmid (p3146) were also co-transformed with plasmids containing an Hsp104 promoter (*HSE*) driving either empty vector (EV; p3168), wildtype Hsp104 (Hsp104^OE^; p3169), Hsp104 with a A503V mutation (Hsp104^A503V^; p3170), or Hsp104 with a A503S mutation (Hsp104^A503S^; p3171).

Yeast strains were grown using standard media and cultivation procedures (Sherman et al., 1986). Nutrient rich media (YPD) or synthetic complete media containing the required amino acids and 2% dextrose (SD) was used. Growth of involved fresh transformants patched onto solid selective media and grown overnight at 30°C before inoculating liquid cultures. Liquid cultures were grown to log phase (OD_600_ = 0.4–0.8; approximately 24 h), or until saturation (OD_600_ = 1.6 or higher; approximately 48 h).

### Microscopy

For co-localization experiments, strains were transformed with TTR-eGFP (p3146) and mCherry (p3197) or Hsp104-mCherry (p3172). 3D images were captured with a 63X (oil immersion, N.A. 1.4) objective on a DMI600 Leica inverted microscope using Leica K5 camera and LasX software. For print, images were subjected to 3D deconvolution, using AutoQuant deconvolution software and shown as maximum projection images. For quantifying the number of cells with TTR-eGFP puncta, strains were transformed with TTR-eGFP (p3146) and either empty vector (p3168), Hsp104^OE^ (p3169), Hsp104^A503V^ (p3170), or Hsp104^A503S^ (p3171). Strains were grown to log or saturation, and 3D images of independent transformants were captured and quantified for the number of cells that contained GFP puncta compared to all cells expressing GFP.

### Biochemical analysis of yeast lysates

Cell lysates were prepared from 50 ml cultures by glass bead lysis with 1x lysis buffer (50 mM Tris–HCl, 100 mM KCl, 20 mM, MgCl_2_•6H_2_0, 2 mM EDTA, 10% glycerol; pH 7.5), protease inhibitor cocktail (Sigma) and PMSF (Sigma), while kept on ice to prevent protease degradation, as described in Sharma et al. (2017). To analyze protein steady state levels, 75–125 μg of crude cell extracts were treated with 2% SDS sample buffer (25 mM Tris pH 6.8, 200 mM glycine, 5% glycerol, and 0.025% bromophenol blue) and boiled for 8 min at 98°C before electrophoretically resolved on 10% acrylamide gel. Gels were transferred to PVDF membrane, blocked using Tropix® I-Block™ solution (Applied Biosystems, Ref: T2015) before antibody exposure (Supplementary Table 4). Blots were subjected to standard Western blot procedures. Given signal proximity, TTR antibody was detected with horse radish peroxidase using a luminol stubstrate, while PGK antibody was detected with alkaline phosphatase using a CDP-STAR Tropix® substrate. Antibody signal was captured by the Amersham Imager 600 and images quantified by ImageJ. Well-trap assays (Liebman et al., 2006) were performed similarly to above, except unboiled samples were incubated at room temperature for 8 min instead of boiling.

### Sucrose gradient fractionation

Discontinuous sucrose gradients were performed as previously described Dorweiler et al. (2020). Briefly, gradients consisting of 10, 40, and 60% sucrose (w/vol) were made in 1× lysis buffer and stored at-80°C. Gradients were thawed on ice, and an aliquot containing 6–8 mg of protein was loaded onto the gradient. Samples were spun at 16,000× *g* 4°C for 120 min in a swinging bucket rotor. 10 fractions of 180 μl each were collected per sample. Equivalent volumes of fractions were loaded onto SDS-PAGE and subjected to standard Western blot procedures. The intensity of individual fractions was compared to total intensity of combined fractions from each trial to derive a percent value of resolvable protein per fraction. These values per fraction were then binned according to sucrose layer to derive the percent of total protein: fractions 1–4 representing the 10% layer, fractions 5–7 representing the 40% layer, and fractions 8–10 and the pellet representing the 60% layer.

### SDD-AGE

To analyze SDS-resistant oligomers by SDD-AGE, 230 μg of crude lysate, or a 1:1 ratio of sucrose gradient fraction to 1× lysis buffer, was treated with 2% SDS loading buffer and incubated at room temperature for a minimum of 5 min prior to loading into a 1.5% agarose gel containing 0.1% SDS. Protein was transferred to nitrocellulose membrane *via* capillary transfer, as described in Halfmann and Lindquist (2008). Blots were subjected to standard Western blot procedures and probed with polyclonal TTR antibody.

## Results

### Hsp104 colocalizes with visible TTR puncta

**Figure 1:**
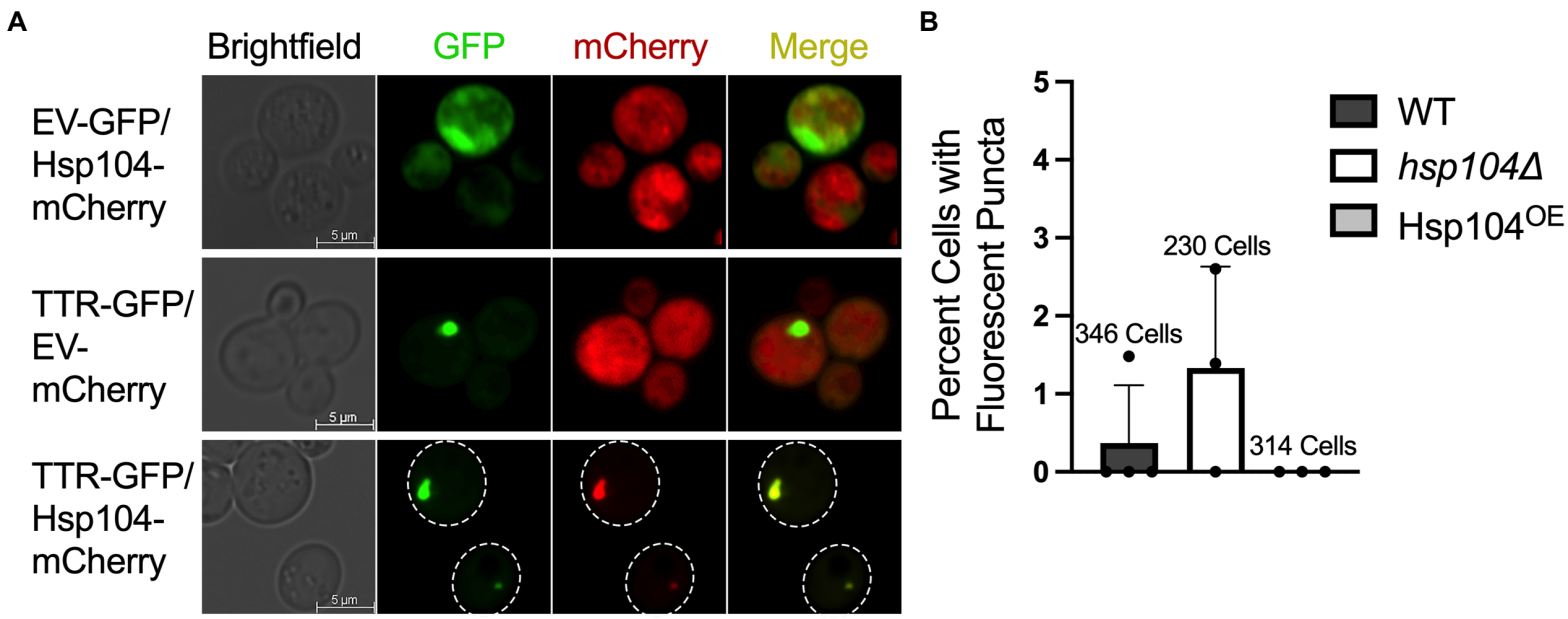
TTR-GFP forms fluorescent puncta that co-localizes with Hsp104. **(A)** *hsp104*∆ cells were transformed with the indicated plasmid combinations and grown for approximately 48 h. Representative fluorescent microscopy images with the indicated proteins are shown. **(B)** Cells in wildtype strains (WT; containing a genomic copy of *HSP104*), *hsp104*∆ strains, and *hsp104*∆ strains with a plasmid moderately overexpressing Hsp104 (Hsp104^OE^) were grown to log (OD_600_ = 0.4–0.8, approximately 24 h). The percentage of GFP expressing cells containing puncta was plotted from 3 independent experiments. Total number of cells counted of the three experiments is reported above. Data is shown as means and standard deviation.

To begin to understand how Hsp104 influences TTR aggregation *in vivo*, we assessed whether Hsp104 co-localized with TTR fluorescent puncta. Using *hsp104*∆ strains, where the only source of Hsp104 was from a plasmid expressing Hsp104-mCherry, TTR puncta consistently colocalized with Hsp104-mCherry in all images (Figure 1A). These data suggest that Hsp104 may potentially interact with TTR-GFP aggregates and influence their formation.

Previous work used visual aggregates to track TTR aggregation, well after cultures reached saturation, reporting approximately 5–7% of cells exhibit TTR-GFP as visible puncta after 72 h of growth (Verma et al., 2018). To determine whether incubation time correlated with the number of cells with visible puncta, we assessed the percent of cells containing puncta in mid-log (OD_600_ = 0.4–0.8) growth after 24 h. Wildtype strains with an endogenous copy of Hsp104 (WT) exhibited on average less than 0.5% of cells with puncta, while strains lacking *HSP104* (*hsp104Δ*) had almost three times as many cells with puncta (Figure 1B) suggesting that Hsp104 appears to play a role in limiting the formation of visible aggregates. In addition, considering results from Verma et al. (2018), the number of cells containing visible TTR puncta appear to increase over time.

It has been shown that increased levels of Hsp104 result in the loss of an endogenous yeast amyloid called the [*PSI*^+^] prion (Chernoff et al., 1995). A plasmid containing wildtype *HSP104* driven by the *HSP104* promoter on a centromeric plasmid increases steady state Hsp104 levels approximately 8-10-fold more when compared to endogenous levels (Supplementary Figure 1). When this same plasmid was introduced in a *hsp104*∆ strain (Hsp104^OE^; see materials and methods), we found that cells did not have any detectable TTR-GFP puncta after 24 h of growth (Figure 1B). It is important to note that the presence or absence of Hsp104 in log cultures does not impact TTR steady state levels (Supplementary Figure 2A), and TTR expression does not impact endogenous or plasmid-derived Hsp104 steady state levels (Supplementary Figures 2B,C). We also verified that these plasmids produced more than endogenous levels of Hsp104 by an independent prion-curing assay, since excess Hsp104 cures the [*PSI*^+^] prion. Using weak [*PSI*^+^] log cultures, we show that the plasmid derived Hsp104^OE^ resulted in 5-fold more [*PSI*^+^] loss compared to strains with empty vector plasmids (Supplementary Figure 3).

### Hsp104 increases the abundance of SDS-resistant TTR populations

**Figure 2:**
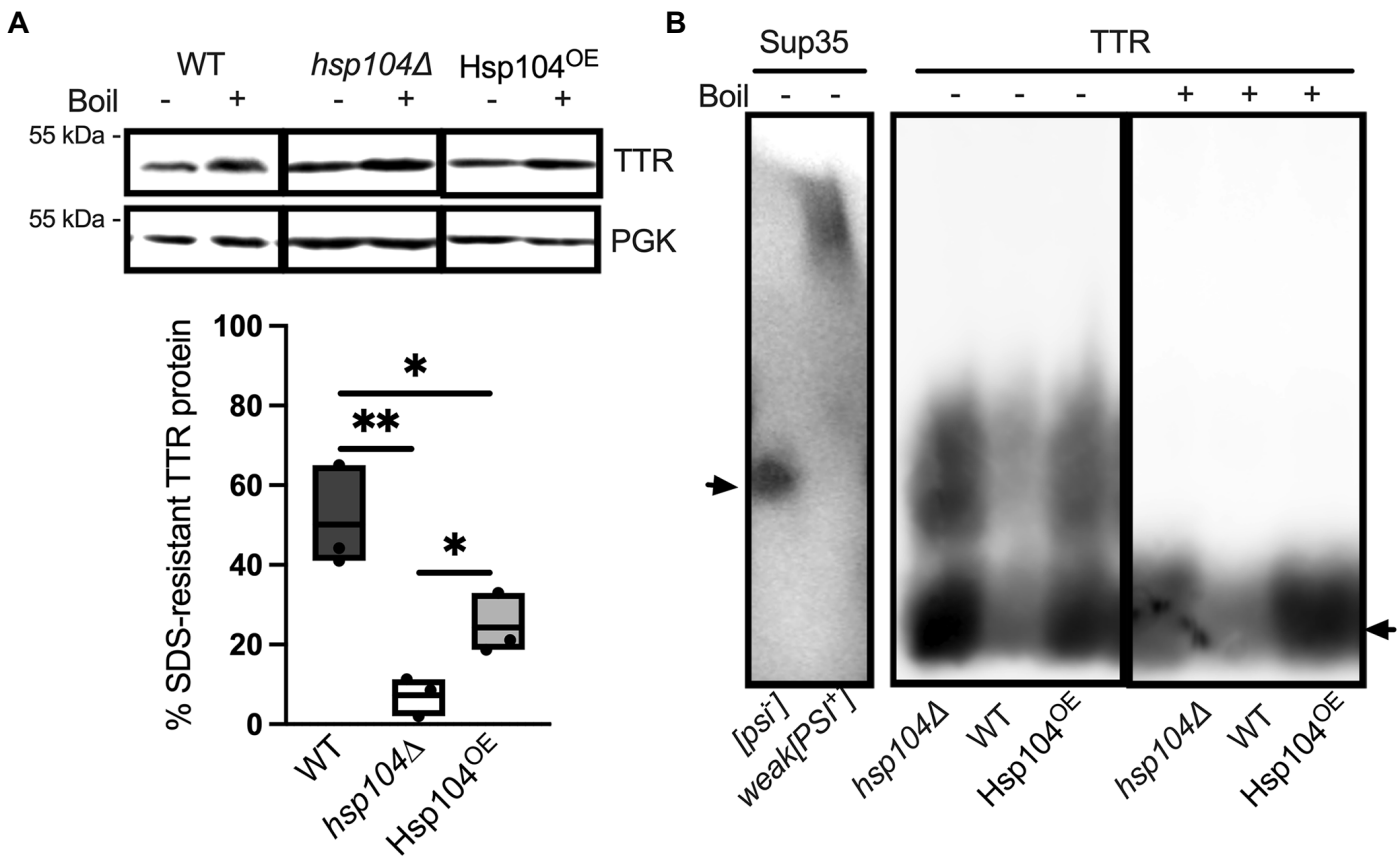
Hsp104 enhances the formation of SDS-resistant populations, but oligomer size appears to be unaffected **(A)** (Top) Lysates from WT or *hsp104*∆ strains co-transformed with TTR-GFP and HSE-EV, or *hsp104∆* strains co-transformed with TTR-GFP and HSE-Hsp104 (Hsp104^OE^) were incubated at room temperature (−) or boiled (+). Representative Western blots using anti-TTR or anti-PGK antibody, captured with different detection methods (see Materials and Methods), is shown. PGK was used to confirm even sample loading and quantification of the amount of SDS-resistant aggregated protein was determined by calculating the ratio of unboiled to boiled sample (Bottom). Quantification is from 3 Independent trials. Data is shown as means and ranges. *p ≤ 0.05, **p ≤ 0.01, by unpaired two-tailed *t*-test. **(B)** SDD-AGE analysis was used to resolve SDS-resistant oligomers. Lysates from [*psi*^−^] or weak[*PSI^+^*] strains (left) or TTR expressing lysates from the indicated (right) were subjected to SDD-AGE analysis and visualized with anti-Sup35 and anti-TTR, respectively. Arrows denote monomeric Sup35 (Left) or TTR-GFP (right). It is important to note that lysates from strains are loaded in a different order than **(A)**. Representative images are shown.

If the low percentage of cells with visible TTR puncta correlates with the amount of aggregated TTR, then biochemical analysis should detect high amounts of soluble monomeric TTR in all strains. Previous work has shown that TTR expressed in yeast forms SDS-resistant aggregates (Verma et al., 2018). Similarly, amyloidogenic proteins are commonly detergent resistant and only enter an SDS-PAGE gel upon boiling (Kazantsev et al., 1999; Kryndushkin et al., 2003; Nizhnikov et al., 2014; Andrich et al., 2017). To determine the percentage of TTR protein that is SDS-resistant, well-trap assays were performed. In this assay, large SDS-resistant aggregates are trapped in the well in unboiled lysates and are broken and resolved as a monomer in boiled lysates. Roughly 50% of TTR from WT log cultures are SDS-resistant (Figure 2A). We also found that a L55P TTR mutation, which has previously considered to be amyloidogenic in yeast (Gomes et al., 2012), as well as a L110Q mutation show similar aggregation compared to wildtype TTR (Supplementary Figure 4). Interestingly, there is a 5-fold reduction of SDS-resistant TTR populations in *hsp104∆,* suggesting that Hsp104 may play a role in facilitating the formation of SDS-resistant populations. Conversely, increased levels of Hsp104 through plasmid expression in Hsp104^OE^ showed a 2-fold reduction in SDS-resistant aggregates compared to WT strains expressing endogenous Hsp104. It is important to note that the amount of TTR that is not visible in unboiled WT strains is well beyond the percentage of cells that contain visible puncta (Figure 1B), indicating that these SDS-resistant populations are likely not visible as fluorescent puncta.

Semi-denaturing agarose gels (SDD-AGE; Kryndushkin et al., 2003; Bagriantsev et al., 2006; Halfmann and Lindquist, 2008) can resolve SDS-resistant oligomer populations that may be too large to enter a SDS-PAGE gel. In the case of the [*PSI*^+^] prion, SDS-resistant oligomers consisting of approximately 9–50 monomers of Sup35 can be detected (Kryndushkin et al., 2003). We find TTR forms SDS-resistant oligomeric species in unboiled wildtype lysates (Figure 2B). These TTR protein smears are resolved closer to the bottom of the gel when compared to weak [*PSI*^+^], indicating that TTR oligomers are smaller than [*PSI*^+^] oligomers. Given that TTR is unable to maintain its tetrameric structure in the presence of SDS (Colon and Kelly, 1992), and little to no dimers or tetramers were detected by SDS-PAGE and Western blots (Supplementary Figure 5), the size range of these smears, as well as their disappearance upon boiling (Figure 2B), suggest TTR forms SDS-resistant oligomers and not tetramers. Interestingly, the SDS-resistant oligomer did not change size when Hsp104 was absent or overexpressed, suggesting that Hsp104 does not impact the formation of these SDS-resistant oligomers.

### TTR aggregate size changes in Hsp104^OE^ strains

**Figure 3:**
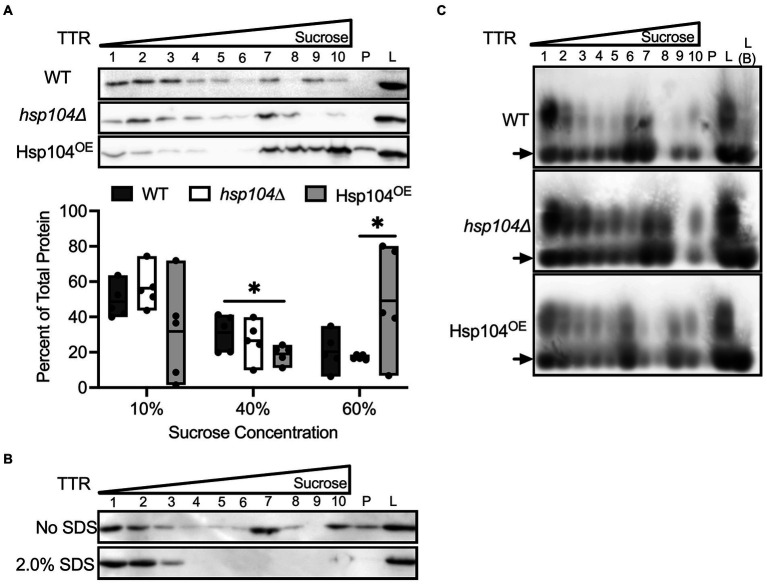
TTR sediments with higher molecular weight complexes in Hsp104^OE^ log cultures. **(A)** Lysates from the indicated strains were loaded onto a discontinuous sucrose gradient (10, 40, 60% sucrose, *w/w*). 10 fractions were collected, with fractions 1–4 equivalent to the supernatant and 10% sucrose, fractions 5–7 equivalent to 40% sucrose, and fractions 8–10 equivalent to the 60% sucrose layers. P indicates pellet and L indicates separately loaded whole cell lysates. (Top) Representative blots of TTR sedimentation in log cultures are shown. (Bottom) Quantification of sedimented TTR signal in fractions 1–4 (10%), fractions 5–7 (40%), or fractions 8-P (60%) compared to total protein in all fractions. All quantification represents 4–6 independent cultures per strain. Data is shown as means and ranges. *p ≤ 0.05 by unpaired two-tailed *t*-test. **(B)** The lysates of WT strain in log growth were either treated in the presence or absence of 2.0% SDS prior to centrifugation on discontinuous sucrose gradients. Gradients were spun at 25°C to prevent SDS precipitation. **(C)** Untreated lysates were subjected to sucrose gradient sedimentation, and lysates were resolved by SDD-AGE and visualized with anti-TTR. Note that L **(B)** indicates crude lysate that were boiled prior to loading. Arrows denote TTR monomers. Representative images are shown.

TTR expression in our yeast model yields both monomeric and aggregated TTR protein. While SDS-treatment shows that oligomers and aggregates are SDS-resistant, it is unclear whether the monomers generated by SDS-treatment are normally associated in higher molecular weight complexes in the absence of SDS. To answer this question, we employed a different approach to assess the general distribution of TTR aggregate sizes in all three strains. A discontinuous sucrose gradient (10, 40, and 60% sucrose, *w/w*) can trap protein complexes and aggregates of different sizes within the interface between different sucrose concentrations (Dorweiler et al., 2020). Crude lysates were directly loaded onto a discontinuous gradient and collected in 10 fractions and subjected to Western blot using anti-TTR antibody (Figure 3A). To quantify protein sedimentation, we combined TTR signal intensity from the top (soluble protein or small size complexes: fractions 1–4; “10%”), middle (moderate size complexes: fractions 5–7; “40%”), and bottom and pellet (high molecular weight complexes; fractions 8–10 and pellet; “60%”; Figure 3A) fractions of the gradient.

**Figure 5:**
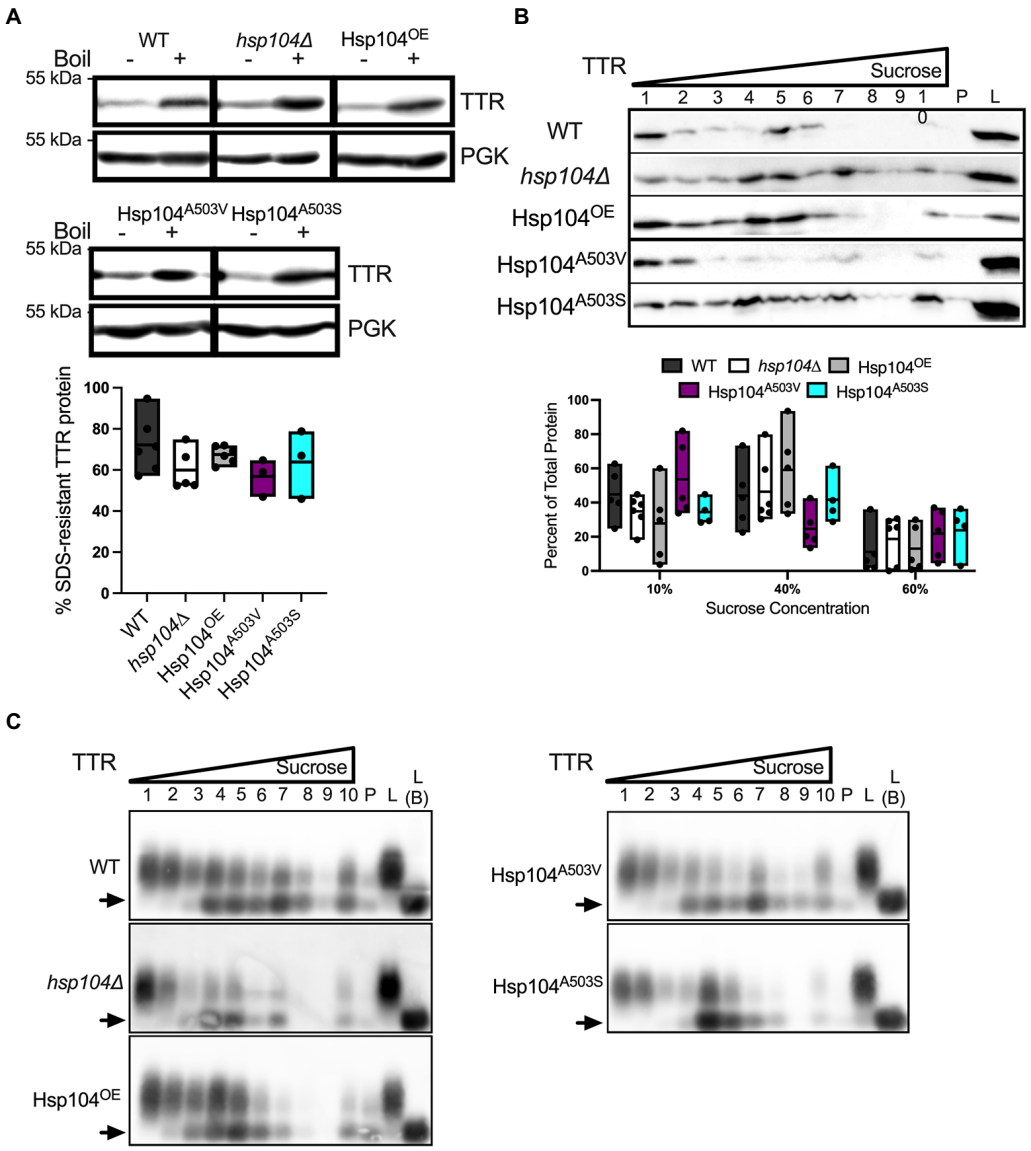
The effect of Hsp104 on aggregating TTR is muted in saturated cultures. All cultures were grown to saturation (OD_600_ ≥ 2.0) over 48 h. **(A)** (Top) Representative blots from lysates were incubated at room temperature (−) or boiled (+). (Bottom) Quantification of SDS-resistant aggregated protein. Quantification is from at least 3 independent trials. Data is shown as means and ranges. **(B)** Lysates subjected to discontinuous sucrose gradient centrifugation and detected with anti-TTR antibody. (Top) Representative blots of TTR sedimentation. (Bottom) Quantification of sedimented TTR signal. All quantification represents 4–6 independent cultures per strain. Data is shown as means and ranges. **(C)** Sucrose gradient fractions of indicated strains from saturated cultures were resolved by SDD-AGE and visualized with anti-TTR. Note that L **(B)** indicates crude lysate that were boiled prior to loading. Arrows denote TTR monomers. Representative images are shown.

In wildtype log cultures, the bulk of sedimented protein is in the 10% fractions (Figures 3A,B). Conversely, there is a large difference in sedimentation patterns between Hsp104^OE^ and the other strains. While WT and *hsp104∆* strains show the bulk of TTR fractionate to 10% (Figure 3A; Supplementary Figure 6A), the overexpression of Hsp104 (Hsp104^OE^) shows TTR preferentially fractionating to either the 10% or 60% fractions (Figure 3A; Supplementary Figure 6B). The inconsistent TTR sedimentation pattern from Hsp104^OE^ strains is unlikely a product of variable Hsp104 expression from the centromeric plasmid since Hsp104 steady state levels appeared to be similar (Supplementary Figure 6B). Closer analysis of individual trials show that an increase in TTR sedimentation in the 60% sucrose fractions is associated with a decrease in 10% sucrose fractions and vice versa (Supplementary Figure 6B). Interestingly, there is no dramatic change in TTR sedimentation in the 40% fractions. Taking the SDS-treatment and sucrose gradient results together, our data show that while SDS-resistant oligomer formation is Hsp104 independent (Figure 5B), the formation of SDS-resistant populations and overall TTR aggregation is influenced by Hsp104 overexpression (Figures 3A, 2A).

To better understand the relationship between SDS-resistant populations, oligomers resolved by SDD-AGE, and TTR aggregates resolved by sucrose gradient, we performed two experiments. In the first experiment, we wanted to determine whether TTR associated with the high molecular weight fractions of a sucrose gradient are involved in SDS-sensitive associations. Based on the relatively smaller size of SDS-resistant oligomers resolved by SDD-AGE, and TTR sedimentation in high molecular weight fractions, we suspected that SDS treatment of crude lysates would dissolve larger SDS-sensitive structures into smaller SDS-resistant oligomers. Therefore, crude lysates were treated with SDS prior to fractionation on the sucrose gradient. SDS-treated crude lysates result in a TTR sedimentation pattern to only the top 3 fractions of the gradient (Figure 3B), suggesting that TTR protein in higher gradient fractions are held together by SDS-sensitive associations. In the second experiment, we wanted to confirm whether SDS-resistant oligomers were found in higher molecular weight TTR fractions. Untreated fractionated lysates were run on SDD-AGE gels. We found that SDS-resistant oligomers are associated with all fractions regardless of sedimentation patterns or Hsp104 levels (Figure 3C). Taken together, these data support an aggregate model comprised of smaller SDS-resistant oligomers held together as higher ordered structures by SDS-sensitive associations.

### Hsp104^A503S^ mutant maintains SDS-resistant TTR oligomer sizes but lead to larger TTR aggregates

The middle domain of Hsp104 has been shown to be important for regulating disaggregase activity and co-chaperone interaction during aggregate disassembly (Schirmer et al., 2004; Lee et al., 2010; Sielaff and Tsai, 2010; Desantis and Shorter, 2012; Dulle et al., 2014; Jackrel et al., 2014). Several mutations within the middle domain of Hsp104 have been shown to reduce aggregation and toxicity associated with aggregated amyloidogenic human protein *in vivo* (Jackrel et al., 2014; March et al., 2020), and potentiate the disaggregase activity with co-chaperones *in vitro* (Jackrel et al., 2014). We asked whether middle domain mutations would also reduce TTR protein aggregation. Middle domain mutants A503V or A503S in a *hsp104∆* background, to create Hsp104^A503V^ and Hsp104^A503S^ strains, were tested. Steady state levels of these Hsp104 mutants were similar to Hsp104^OE^ strain at 8–10 times higher concentration than endogenous levels (Supplementary Figure 1). Similarly, TTR steady state levels and the percentage of cells exhibiting TTR puncta were similar in Hsp104^OE^, Hsp104^A503V^, or Hsp104^A503S^ log cultures (Supplementary Figures 2A, 7A).

**Figure 4:**
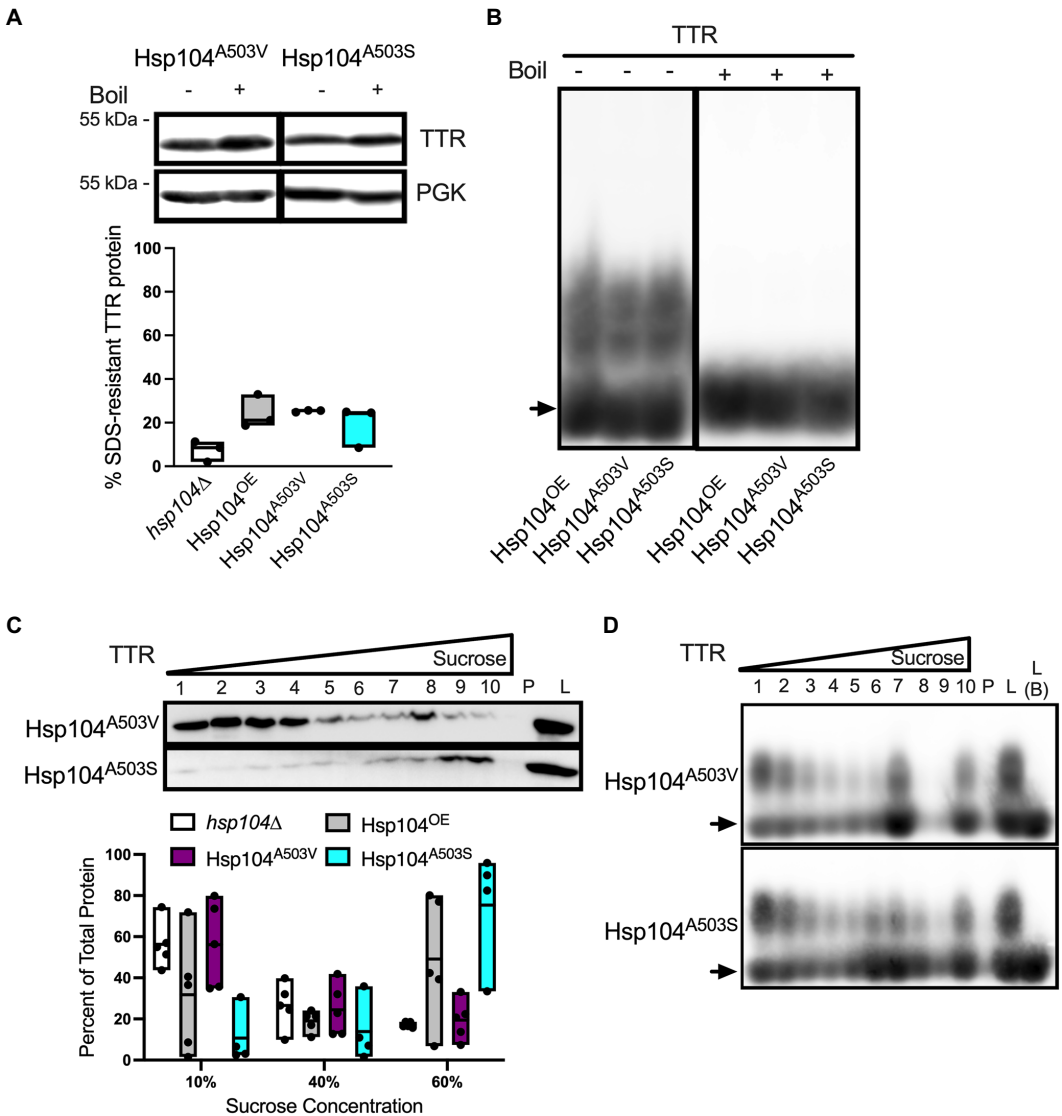
Strains expressing Hsp104^A503V^ show different sedimentation profiles compared to strains expressing Hsp104^OE^. **(A)** Similar to Figure 2A, TTR expressing *hsp104∆* strains co-transformed with either Hsp104^OE^, Hsp104^A503V^, or Hsp104^A503S^. (Top) Representative Western blots. (Bottom) Quantification is from 3 independent trials. *hsp104∆* and Hsp104^OE^ data from Figure 2A. Data is shown as means and ranges. **(B)** Log culture lysates of indicated strains were resolved by SDD-AGE. The arrow denotes TTR monomers. Representative images are shown. **(C)** Western blot of lysates from the indicated log strains subjected to a discontinuous sucrose gradient. (Top) Representative blots of TTR sedimentation from lysates, and (Bottom) quantification of TTR signal as percent of total protein, as in Figure 3A. *hsp104∆* and Hsp104^OE^ data from Figure 3A. All quantification represents 4–5 independent cultures per strain. Data is shown as means and ranges. **(D)** Sucrose gradient fractions of indicated strains were resolved by SDD-AGE as in Figure 3B. Arrows denote TTR monomers. Representative images are shown.

Well-trap and SDD-AGE experiments show that the Hsp104^A503V^ and Hsp104^A503S^ mutants show similar percent SDS-resistant TTR populations and size of SDS-resistant TTR oligomers compared with Hsp104^OE^ (Figures 4A,B). Sucrose gradient fractionation of TTR in Hsp104^A503V^ sedimented similarly to *hsp104∆* strains with the bulk of the protein found in 10% fractions. Interestingly, Hsp104^A503S^ shows strong sedimentation of TTR to the bottom of the gradient, much more consistently than Hsp104^OE^. The dichotomy of how each Hsp104 mutant sediments TTR populations, despite maintaining TTR oligomer sizes in all fractions (Figure 4D), suggests a functional difference between the two variants.

### Hsp104 related changes to TTR aggregation are not observed in saturated cultures

Since visible TTR aggregates are more abundant in saturated cultures (Verma et al., 2018) compared to log cultures (Figure 1B), we asked how TTR aggregation changes when cultures are in saturation. Cells in saturated culture have slower cell cycles, increased chronological aging, undergo metabolic shifts in response to diminishing nutrient availability, and have reduced ATP (Bilinski et al., 2017; Zimmermann et al., 2018). In saturated cultures, percentage of cells with fluorescent puncta increased in both WT and *hsp104Δ* strains compared to log cultures (Supplementary Figure 7), confirming that the number of cells with visible aggregates increase with time. Surprisingly, increasing wildtype or mutant Hsp104 levels did not impact the number of cells with fluorescent puncta (Supplementary Figure 7B), suggesting that any potential effect that Hsp104 overexpression has on puncta formation in log cultures is lost in saturation. Interestingly, Hsp104^OE^ does not seem to be completely inactive in saturated cultures since a slight but significant increase in prion loss is observed when compared to log cultures (Supplementary Figure 3). However, saturated cultures showed decreased plasmid derived Hsp104 steady state levels in the presence of TTR (Supplementary Figures 2B,C) and a modest increase in cell death when compared to log cultures (Supplementary Figure 8).

The aggregation state of TTR in saturated cultures showed no difference when comparing the percent of protein in SDS-resistant aggregates (Figure 5A) or the size of oligomers (Supplementary Figure 9) between strains. Similarly, sucrose gradient sedimentation showed TTR fractionating to the top two-thirds of the sucrose gradient in all strains (Figure 5B). SDS-resistant oligomers persist in all sucrose gradient fractions (Figure 5C), similar to log cultures. However, while log cultures showed monomeric protein found in fractions 1–3 in all strains (Figures 3D, 4D), the monomeric protein was absent in saturated cultures in these fractions (Figure 5C).

Nutrient availability declines in saturated cultures due to exhaustion of resources and buildup of waste material. To ensure that our observed TTR results from saturated cultures was not a simple product of limited nutrients, we refreshed the culture media every six hours between reaching mid-log growth to 48 h. After 48 h, we found that refreshing media had no effect on the percentage of TTR in SDS-resistant aggregates (Supplementary Figure 10). Taken together, our saturation data indicates that aggregating TTR populations change between log and saturated cultures, but TTR aggregates do not respond to altered levels of Hsp104 in saturated cultures.

## Discussion

**Figure 6:**
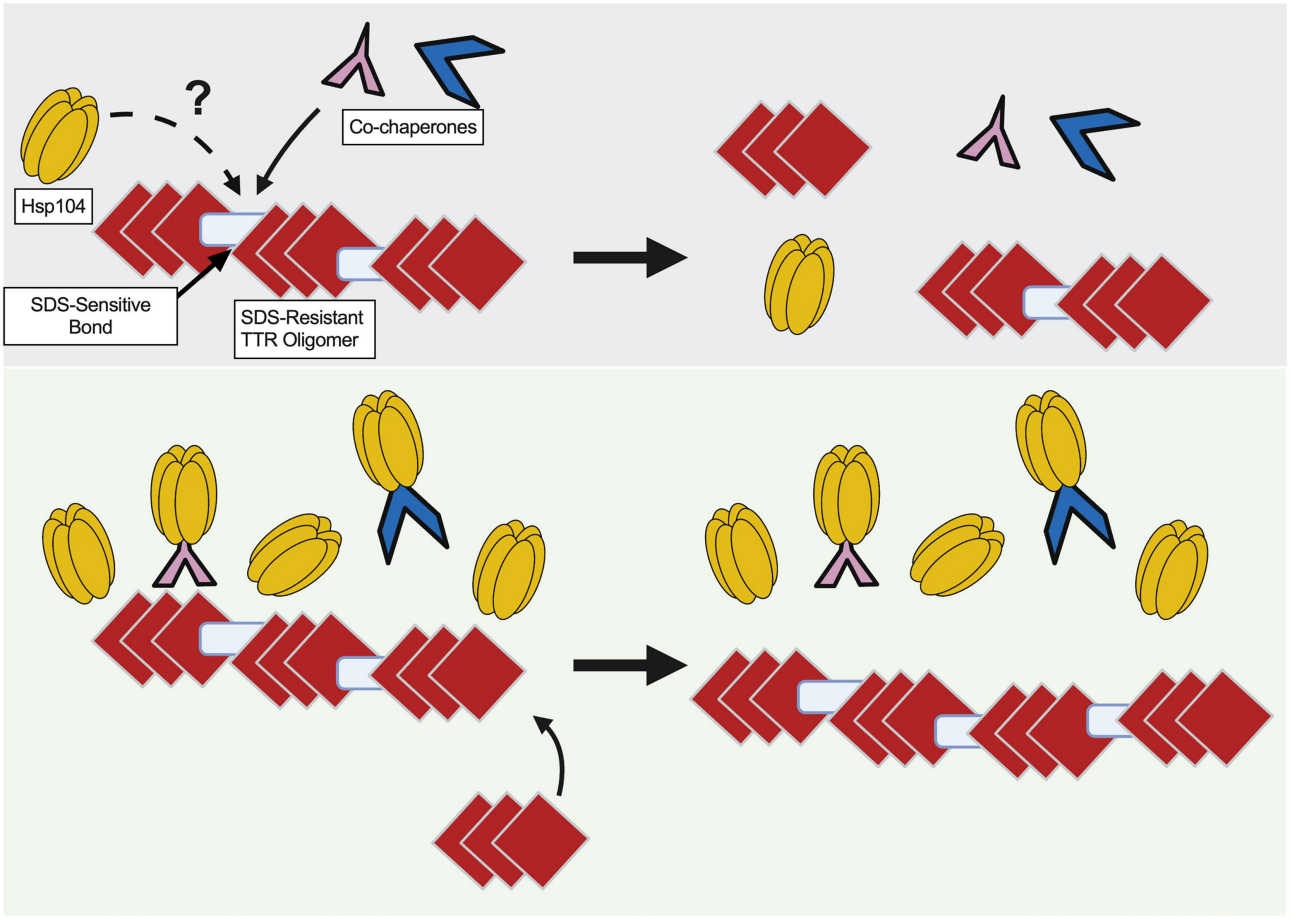
Model of TTR aggregate growth by excess Hsp104. Red diamonds represent SDS-resistant TTR oligomers and blue rectangles represent SDS-sensitive associations to depict large TTR aggregates. (Top) Co-chaperones, possibly along with Hsp104, play a role in maintaining smaller sized aggregates. (Bottom) Excess Hsp104, or Hsp104^A503S^, increases aggregate size by possibly titrating chaperones away from the TTR aggregate.

Here, we used a yeast-based system to understand TTR aggregate formation and how the molecular disaggregase, Hsp104, influences this process. Our study shows that fluorescently labeled Hsp104 colocalizes with visible TTR puncta (Figure 1A), but fluorescent puncta are not fully representative of TTR aggregation (Figures 1B, 2A). Instead, this co-localization between TTR and Hsp104 suggest a molecular interaction *in vivo* and is the impetus for studying whether altering levels of Hsp104 influences aggregating TTR. Increased amount of SDS-resistant TTR protein form in the presence of Hsp104 (Figure 2A), yet SDS-resistant TTR oligomers and higher order structures containing SDS-sensitive associations form independently of Hsp104 (*hsp104Δ*, Figures 2B,3A, B). These results suggest that the structure of higher order TTR aggregates is comprised of smaller SDS-resistant oligomeric populations held together by SDS-sensitive bonds (Figure 6). Introduction of the Hsp104^A503S^ mutant shows TTR aggregates consistently sediment in higher molecular weight fractions (Figure 4C), which is consistent with previous studies that show Hsp104 middle domain mutations are more influential in the formation of human amyloidogenic protein aggregates compared to wildtype Hsp104 (Jackrel et al., 2014; March et al., 2020). Lastly, our work shows that Hsp104 exerts an effect on TTR aggregate populations during log phase growth, but not in saturated cultures. Given that media replacement does not change SDS-resistant TTR populations in saturated conditions (Figure 5A; Supplementary Figure 10), it is possible that the inability to respond to elevated Hsp104 levels is due to proteostasis collapse (Labbadia and Morimoto, 2015; Klaips et al., 2018).

### TTR aggregate organization in yeast

Amyloid is commonly resistant to detergent solubilization (Kazantsev et al., 1999; Kryndushkin et al., 2003; Nizhnikov et al., 2014; Andrich et al., 2017) and therefore can be used to detect whether a protein aggregate is in an amyloidogenic state. Using well-trap assays, we show that a portion of the TTR population is in large SDS-resistant populations. Further analysis using semi-denaturing gels (SDD-AGE) shows both oligomeric and monomeric protein are detected. Tetrameric TTR-GFP is approximately 160 kDa but is unlikely to be stable in the presence of 2% SDS (Colon and Kelly, 1992). Little or no high molecular weight TTR signal is detected in our SDS-PAGE gels (Supplementary Figure 5). Given the SDS-PAGE system is able to resolve proteins under 400 kDa, the observation of larger molecule weight smears by SDD-AGE indicate a SDS-resistant TTR population larger than the tetramer.

### The formation of the TTR aggregate and the role of Hsp104

Hsp104’s fragmentation activity has been well characterized in its role in prion propagation by fragmenting aggregates and generating smaller transmissible particles that can be inherited by daughter cells (Chernoff et al., 1995; Paushkin et al., 1996; Shorter and Lindquist, 2004; Satpute-Krishnan et al., 2007). Overexpression of Hsp104 leads to prion loss, however the mechanism of how overexpressed Hsp104 acts on the prion aggregate is still under debate. There are two prominent models that can explain prion loss: trimming and malpartition. The trimming model suggests that excess Hsp104 facilitates the disassembly of the prion aggregate fibril from the ends (Zhao et al., 2017; Greene et al., 2020). This model is largely supported by fluorescently labeled prion protein and the observed changes in generated fluorescent puncta (Zhao et al., 2017). In the malpartition model, aggregates become so large they are asymmetrically retained in the mother cell, unable to pass through the bud neck (Ness et al., 2017; Greene et al., 2020). It has been suggested that excess Hsp104 results in aggregate stability (Ness et al., 2017). Conversely, Hsp104 overexpression could unbalance the proper co-chaperone stoichiometric ratio required to form functional Hsp104/70/40 complexes, thereby decreasing fragmentation necessary for prion propagation (Winkler et al., 2012; Naeimi and Serio, 2022).

We show that TTR aggregates sediment in higher molecular weight fractions occasionally with Hsp104^OE^ (Figure 3A) and consistently with Hsp104^A503S^ (Figure 4C). It is important to recognize that SDS-resistant oligomers form in the absence of Hsp104 (Figure 2B), and TTR aggregate sedimentation patterns are similar between *hsp104∆* and WT strains (Figure 3A). Therefore, while Hsp104 appears to co-localize with visible aggregates (Figure 1), it is unclear whether the chaperone is needed for aggregate formation, remodeling, or maintenance. In the case of TTR aggregates, it is possible that co-chaperones such as Hsp70 and Hsp40 work independently from Hsp104. This idea is supported by *in vitro* studies that show homologous human chaperone combinations disassemble and remodel human aggregating amyloidogenic proteins in the absence of Hsp104 (Duennwald et al., 2012; Scior et al., 2018; Franco et al., 2021).

Overexpression of Hsp104^A503S^ results in the sedimentation of TTR to the lowest fractions of the gradient (Figure 4C), suggesting that excess Hsp104^A503S^ increases aggregate size. This increase in size is consistent with the malpartition model. It has been shown that this potentiated Hsp104 variant has increased ATP hydrolysis, has activity independent of the Hsp70 and altered substrate translocation (Jackrel et al., 2014; Durie et al., 2019). Given the relationship between excess Hsp104^A503S^ and increased TTR aggregate size, it is possible that the middle domain mutant may impact co-chaperones interactions that are required for maintaining smaller aggregate populations in log cultures (Figure 6).

In humans, the TTR monomer assembles into small oligomers and larger aggregates. These small oligomeric species are toxic (Reixach et al., 2004; Dasari et al., 2019) and have implications associated with poor patient outcomes. We show that in yeast, TTR forms both SDS-resistant oligomeric species and large aggregates, and overexpression of Hsp104^A503S^ appears to shift the equilibrium of TTR to larger aggregates. This shift suggests that chaperone titration may be a novel approach to ameliorating cells of toxic TTR oligomers and delay disease progression. Future studies focusing on the relationship between chaperones and their ability to shift aggregate populations away from oligomeric species may hold promise for therapeutic interventions to combat the onset and progression of ATTR, and potentially other fatal neurodegenerative diseases.

## Data availability statement

The raw data supporting the conclusions of this article are included in the article/supplementary material, further inquiries can be directed to the corresponding author.

## Author contributions

ASK and ALM contributed to the conceptual design of the study, ASK, EED, HEB, JED, LEF, and ALM contributed to the acquisition, analysis, and/or interpretation of the data, and ASK and ALM have written the manuscript.

## Funding

This work was supported by the Greater Milwaukee Foundation (20172483) and the National Institutes of Health (NIH) grant (GM131365) to AM, and the Schmitt Leadership Fellowship to AK.

## Conflict of interest

The authors declare that the research was conducted in the absence of any commercial or financial relationships that could be construed as a potential conflict of interest.

## Publisher’s note

All claims expressed in this article are solely those of the authors and do not necessarily represent those of their affiliated organizations, or those of the publisher, the editors and the reviewers. Any product that may be evaluated in this article, or claim that may be made by its manufacturer, is not guaranteed or endorsed by the publisher.
