# Supplementary material for: The yeast molecular chaperone, Hsp104, influences transthyretin aggregate formation
Source: Front Mol Neurosci. 2022 Dec 16;15:1050472. doi: 10.3389/fnmol.2022.1050472 (PMC9802906; doi:10.3389/fnmol.2022.1050472)
Supplement: Supplementary file 1 [file Data_Sheet_1.docx]

The yeast molecular chaperone, Hsp104, influences Transthyretin (TTR) aggregate formation.

Supplemental materials

**Authors:** Adam S. Knier^1^, Emily E. Davis^1^, Hannah E. Buchholz^1^, Jane E. Dorweiler^1^, Lauryn E. Flannagan^1^, and Anita L. Manogaran^1^*.

1 Department of Biological Sciences, Marquette University, Milwaukee, WI 53233, USA

* Corresponding Author

**Supplemental Methods**

*Cell Viability and Flow Cytometry*.

Cell viability, as determined by resistance to propidium iodide (PI) staining, was assayed using flow cytometry to identify proportion of PI-stained cells within individual cultures. Selective media was inoculated from patched colonies and grown at 30˚C with orbital agitation to appropriate OD. The supernatant was replaced by centrifuging 400 µl sample aliquots for 2 minutes at 2800 rpm and resuspending cell pellet with PBS. Parallel cultures in PBS were boiled at 98˚C for 10 minutes and cooled to room temperature before continuing. PI was added to all cell aliquots at a final concentration of 5µg/ml and placed on ice in the dark for 15 minutes. Samples were diluted with distilled water and run through Beckman Coulter Cytoflex Flow Cytometer. The ECD channel (610/20 BP) was used to measure PI staining. Boiling samples enabled clear identification of dead cell populations and facilitated gating to discern a relative proportion of culture populations that are non-viable/dead. Toxicity was calculated from number of events corresponding to identified dead cells/total number of events captured.

*[PSI^+^] Curing by Hsp104 Overexpression*

The nonsense mutation *ade1-14* in the 74-D694 strain (Chernoff et al., 1995) was used to identify the [*PSI*^+^] state in weak [*PSI^+^*] strains transformed with plasmids containing either wildtype Hsp104 driven by the *HSP104* promoter (Hsp104^OE^) or no gene driven by the *HSP104* promoter (EV). In [*psi^-^*] strains, the Sup35 protein is soluble resulting in translation termination of the *ade1-14* nonsense codon. This premature stop results in a nonfunctional Ade1 protein and accumulation of adenine biosynthetic pathway intermediate, which results in red pigment and red colony color on rich media. In [*PSI*^+^] strains, the majority of the Sup35 protein is aggregated, resulting in readthrough of the nonsense mutation. This readthrough results in a functional Ade1 protein, and white colony color on rich media. Transformants were immediately inoculated into selective liquid media and cell growth was monitored by OD for desired concentration, as described in Methods. Log growth cultures were repeatedly diluted so that the cultures were never allowed to reach saturation. Cultures were plated onto YPD media at desired OD. Prion loss was assessed by the number of red colonies.

*Filter trap assay*

Approximately 50 ug of crude lysate were serially diluted in lysis buffer. Samples were filtered on a dot-blot filtration unit (Dot blot 10383138; Schleicher & Schuell, Inc.) through 0.2 um Nitrocellulose membrane, and washed three times with 1X TBS. Membranes were stained with 1X ponceau stain to determine loading control. Ponceau stains was washed with water, and membrane was blocked for 1 hour. Membrane was subjected to standard Western blot procedure using anti-TTR (Santa Cruz) antibody.

*Nutrient Availability by Media Refresh*

To ensure ample nutrient availability and ATP generation, media was refreshed every 6 hours. Briefly, strains were grown to mid-log phase (approximately 24 hours), pelleted by low-speed centrifugation, and 80% of the media was replaced. This process was repeated every 6 hours until cultures reached 48 hours of growth. Optical densities were taken before pelleting and after media replacement were monitored to ensure that cells were not lost during media replacement. After 48 hours, cultures were lysed and subjected to Western Blot, as described in Materials and Methods. Uneven PGK signal between unboiled vs. boiled samples were consistently observed in these refreshing experiments compared to cells growth continually for 48 hours. We believe this is attributed to the high protein OD (media refresh had approximately OD_600_ = 5.0, compared to continual growth had approximately OD_600_ = 2.0). To compensate for an uneven loading control, the TTR signal was first normalized to respective PGK signal of individual lanes to produce a normalized TTR signal value. The unboiled normalized TTR signal value was compared to the boiled normalized TTR signal to derive the percent of SDS-resistant TTR protein per lysate.

**Supplementary Tables**

Supplementary Table 1 – Yeast Strains used in this study

| Yeast Strain | Name | Genotype | Reference |
| --- | --- | --- | --- |
| 74-D694 | D112 | *ade1*-14 *ura3*-52 *leu2*-3,112 *trp1*-289 *his3*-200 Weak [*PSI^+^*][*pin*^-^] | (Derkatch et al., 1996) |
| 74-D694 | D230 | *ade1*-14 *ura3*-52 *leu2*-3,112 *trp1*-289 *his3*-200 [*psi^-^*][*pin*^-^] | (Chernoff et al., 1995) |
| 74-D694 | M515 | *ade1*-14 *ura3*-52 *leu2*-3,112 *trp1*-289 *his3*-200 hsp104::HIS3 [*psi*^-^][*pin*^-^] | This study |
| 74-D694 | M248 | *ade1*-14 *his3*-200 *trp1*-289 *ura3*-52 *leu2*-3112 HSP104GFP::KANMX6 [*psi*^-^] | (Huh et al., 2003) |

Supplementary Table 2 – Primers used to generate strain M515

| ID | Name | Sequence (5’-3’) |
| --- | --- | --- |
| AM378 | Hsp104_Sense_disruption (His) | AAAGAAATCAACTACACGTACCATAAAATATACAGAATATATGACAGAGCAGAAAGCCCTAGTAAAGC |
| AM379 | Hsp104_Anti_disruption (His) | CTGATTCTTGTTCGAAAGTTTTTAAAAATCACACTATATTAAACTACATAAGAACACCTTTGGTGG |
| AM380 | Hsp104_Diagnostic | TACCCTTGAATCGAATCAGC |

Supplementary Table 3 – Plasmids used in this study

| ID | Name | Yeast Marker | Name in manuscript | Reference |
| --- | --- | --- | --- | --- |
| p3141 | pAG426GPD-ccdB-EGFP | URA3 (2 micron) | EV-GFP | Addgene plasmid # 14204 |
| p3146 | pAG426GPD-TTR-WT-EGFP | URA3 (2 micron) | TTR-GFP | This study, modified from (Derkatch et al., 2004) |
| p3168 | pRS315HSE promoter | Leu 2, CEN | EV | (Jackrel et al., 2014) |
| p3169 | pRS315HSE-HSP104^OE^ | Leu 2, CEN | Hsp104^OE^ | (Jackrel et al., 2014) |
| p3170 | pRS315HSE- HSP104^A503V^ | Leu 2, CEN | Hsp104^A503V^ | (Jackrel et al., 2014) |
| p3171 | pRS315HSE- HSP104^A503S^ | Leu 2, CEN | Hsp104^A503S^ | (Jackrel et al., 2014) |
| p.3172 | pAG415GPD-Hsp104-mCherry | Leu 2, CEN | Hsp104-mCherry | (Malinovska et al., 2012) |
| p.3197 | pAG415GPD-ccdB-mCherry | Leu 2, CEN | EV-mCherry | (Malinovska et al., 2012) |

Supplementary Table 4 – Antibodies used in this study

| Antibody | Dilution | Clonality | Vendor | Identifier |
| --- | --- | --- | --- | --- |
| Pre-albumin (SDS-PAGE) | 1:1000 | Monoclonal | Santa Cruz Biotechnology | Cat # sc-377517 |
| TTR 1-147  (SDD-AGE) | 1:1000 | Polyclonal | Invitrogen | Cat # PA5-27220 |
| Hsp104 | 1:2000 | Polyclonal | Enzo Life Sciences | Prod. No. ADI-SPA-1040 |
| Phosphoglycerate Kinase | 1:1000 | Monoclonal | Novex by Life Technologies | Cat # 459250 |
| Actin | 1:1000 | Monoclonal | Invitrogen | Cat # MA1-744 |
| Green Fluorescent Protein | 1:5000 | Monoclonal | Sigma | Cat # G1546 |
| Anti-Mouse, AP | 1:10,000 | N/A | Sigma Life Sciences | SKU A3562-.5ML |
| Anti-Mouse, HRP | 1:10,000 | N/A | Sigma Life Sciences | SKU A9044-2ML |
| Anti-Rabbit, HRP | 1:10,000 | N/A | Sigma Life Sciences | SKU A9169-2ML |

**Supplemental figure 1. Hsp104^OE^, Hsp104^A503V^, and Hsp104^A503S^ expression is 8-10 times higher than endogenous Hsp104 levels.** A wildtype strain with endogenous GFP tagged Hsp104 was transformed with either an empty vector (EV), wildtype (Hsp104^OE^), or middle domain mutant of Hsp104 (Hsp104^A503V^ or Hsp104^A503S^) driven by the native Hsp104 promoter. The ratio of plasmid-derived Hsp104 expression to endogenous Hsp104 expression is quantified from 3 separate trials. Data is shown as means and standard deviation. *p$\leq0$.05 by unpaired two-tailed t-test.

**Supplemental Figure 2. Saturated strains expressing TTR result in lower plasmid derived Hsp104 levels. (A)** Quantification of TTR levels, normalized to a PGK antibody control, from lysates of the indicated strains. Experiments from log cultures (left) and saturated cultures (right) were done in triplicate. Data is shown as means and standard deviation. *p $\leq$ 0.05, **p ≤ 0.01 by unpaired two-tailed t-test. **(B)** Quantification of endogenous GFP-tagged Hsp104 levels normalized to PGK antibody in strains either containing the GPD-TTR plasmid (+) or the GPD-EV plasmid (-). Anti-GFP antibody was used to detect endogenous Hsp104-GFP integrated gene. Experiments from log cultures (left) and saturated cultures (right) were performed in triplicate. Data is shown as means and standard deviation. **(C)** Quantification of Hsp104 levels from introduced plasmids (Hsp104^OE^) with and without GPD-TTR. Hsp104 antibody was used to detect the plasmid-supplied levels of Hsp104. Experiments from log cultures (left) and saturated cultures (right) were performed in triplicate. Data is shown as means and standard deviation. *p$\leq0$.05 by unpaired two-tailed t-test. Note HSE-Hsp104 is the presence of the plasmid containing Hsp104^OE^ driven by the Hsp104 promoter.

**Supplemental Figure 3. [*PSI*^+^] loss is observed in Hsp104^OE^ strains.** Weak [*PSI^+^*] strains transformed with plasmids containing wildtype Hsp104 driven by the *HSP104* promoter (Hsp104^OE^) or no gene driven by the *HSP104* promoter (EV). Prion loss was assessed by the number of red colonies (see materials and methods). Experiments from log cultures (circle) and saturated cultures (square) were performed in triplicate (approximately 500-900 colonies total) and quantified as percent colonies cured of [*PSI*^+^]. Data is shown as means and ranges. **p ≤ 0.01, ###p ≤ 0.001 by unpaired two-tailed t-test.


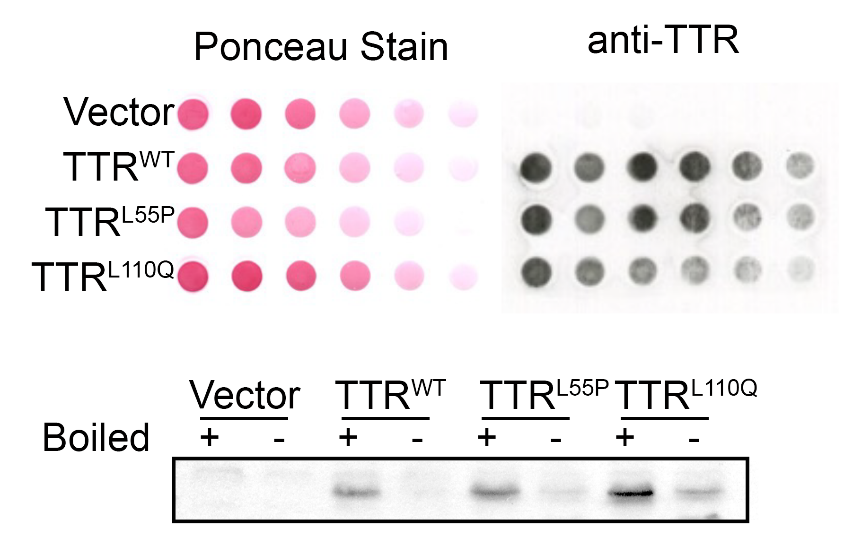


**Supplemental Figure 4. Wildtype TTR and mutant TTR all display protein aggregation. (**Top) Lysates of strains expressing empty vector, wildtype TTR (TTR^WT^), TTR containing a L55P mutation (TTR^L55P^) or TTR containing a L110Q mutation (TTR^L110Q^) were spotted in serial dilution in a filter trap assay. Left is ponceau staining indicating protein trapped on filter, and right is blot incubated with TTR antibody. (Bottom) Well-trap assay for indicated strains, using TTR antibody.

**Supplemental Figure 5. Only monomeric protein is resolved by SDS-PAGE/Western blot.** Strains containing no plasmid (as indicated) were grown to log phase, and Hsp104^OE^ strains were grown to log (L; 24 hours) or saturated (S; 48 hours). Lysates were run on SDS-PAGE and probed with anti-TTR antibody. Untreated samples (-; room temperature) and boiled samples (+; 98^o^C) were incubated for 8 minutes prior to loading.

**Supplemental Figure 6.** **TTR sedimentation profiles in log strains expressing Hsp104^OE^.** The distribution of total percent TTR sedimentation from log culture WT (A) and Hsp104^OE^ (B) Left, strains in 60% sucrose gradient fractions is inversely proportionally with the distribution in 10% fractions. Data is as Figure 3A. Each color series indicates an independent experiment. Right, Crude lysate lanes from sucrose gradient Western blots from A probed with Hsp104 antibody to quantify signal. Hsp104 is normalized to its respective actin signal.

**Supplemental Figure 7. Hsp104 overexpression reduces the percentage of visible puncta only in log cultures. (A)** Fluorescent microscopy of *hsp104*∆ log culture strains, co-transformed with TTR-GFP, and Hsp104^OE^, Hsp104^A503V^, or Hsp104^A503S^. Data taken from 3 or 4 independent transformants and shown as means and standard deviation. **(B)** Similar to A, but strains were grown for 48 hours (saturated cultures). Data taken from 4 independent transformants and shown as means and standard deviation.

**Supplemental Figure 8. Hsp104 overexpression and cell viability in log and saturated cultures.** Wildtype and *hsp104*∆ cells, co-transformed with plasmids for TTR-GFP or empty vector-GFP (EV) and wildtype Hsp104 or EV, stained with propidium iodide to identify dead cells by flow cytometry. EV/EV denotes a wildtype strain co-transformed with EV-GFP (no TTR) and HSE-EV (no Hsp104). The percentage of dead cells was determined by comparing samples to gated heat killed (98˚C) populations. The experiment contains at least 3 independent replicates per group. Approximately 10,000 cells counted per trial. Data is shown as means and ranges. *p$\leq$0.05, ****p$\leq$.0001, by unpaired two-tailed t-test.


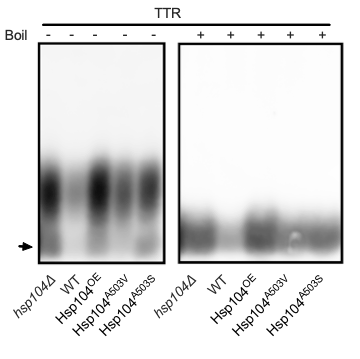


**Supplemental Figure 9. TTR forms SDS-resistant TTR oligomers in saturated cultures.** Lysates from saturated cultures from the indicated strains were subjected to SDD-AGE and visualized with anti-TTR antibody. Arrows denote TTR monomers. Representative images shown.


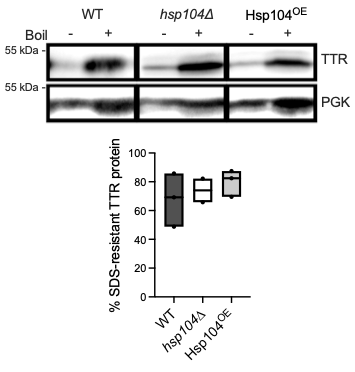


**Supplemental Figure 10. Refreshing the media has no effect on the percent total SDS-resistant TTR protein in saturated cultures.** Strains were grown to mid-log phase (approximately 24 hours). After 24 hours, 80% of the media was replaced every 6 hours for another 24 hours. (Top) Representative blots from lysates incubated at room temperature (-) or boiled (+). (Bottom) Quantification of WT and Hsp104^OE^ is from 3 independent trials while *hsp104∆* is from at 2 independent trials. Data is shown as means and ranges.


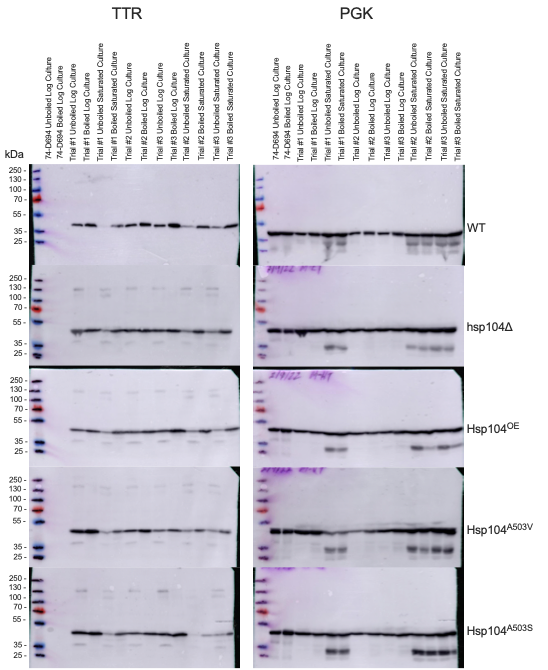


**Supplemental raw blots A. Raw Western blots of well-trap assays. Relevant to Figure 2A, 4A, 5A, and Supplemental Figures 2A, 4.** Note that TTR antibody is using at HRP luminol detection system and PGK is using an AP-CDPstar detection system.

**
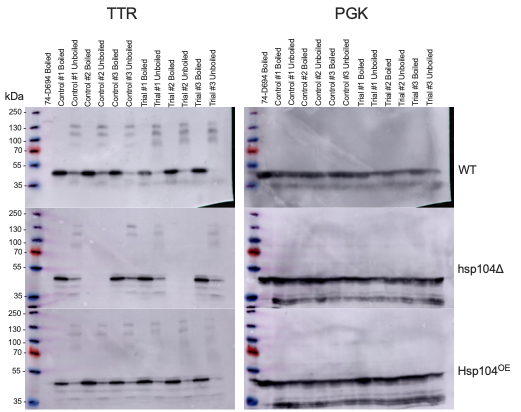
**

**Supplemental raw blots B. Raw Western blots of well-trap assays. Relevant Supplemental Figures 9 (media refresh experiments).** Note that TTR antibody is using at HRP luminol detection system and PGK is using an AP-CDPstar detection system.

**
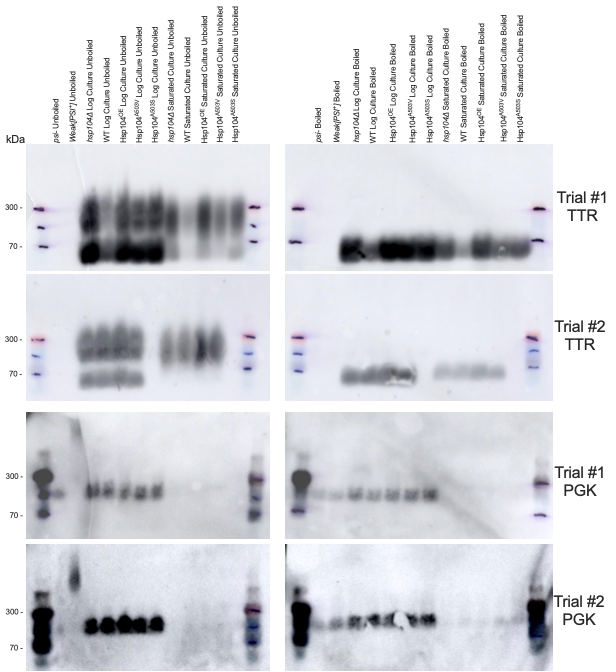
**

**Supplemental raw blot C. Raw SDD-AGE gels of whole cell lysates. Relevant to Figures 2B, 4B, and Supplemental Figure 8.**


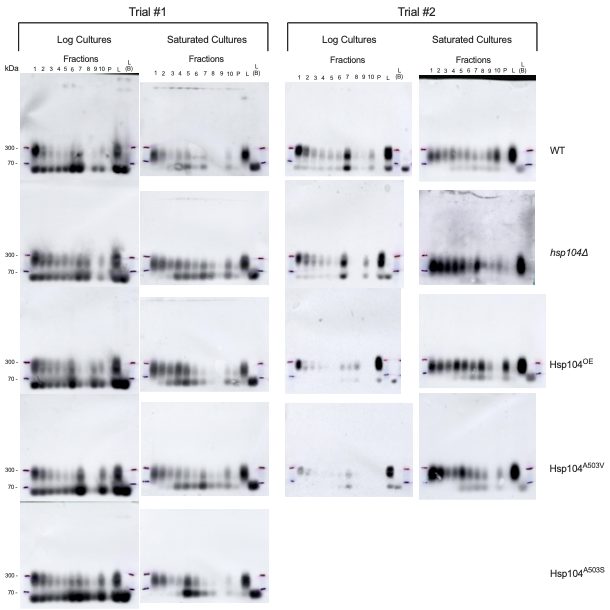


**Supplemental raw blots D. Raw SDD-AGE gels of sucrose gradients. Relevant to Figures 3C, 4D, and 5C.**

**
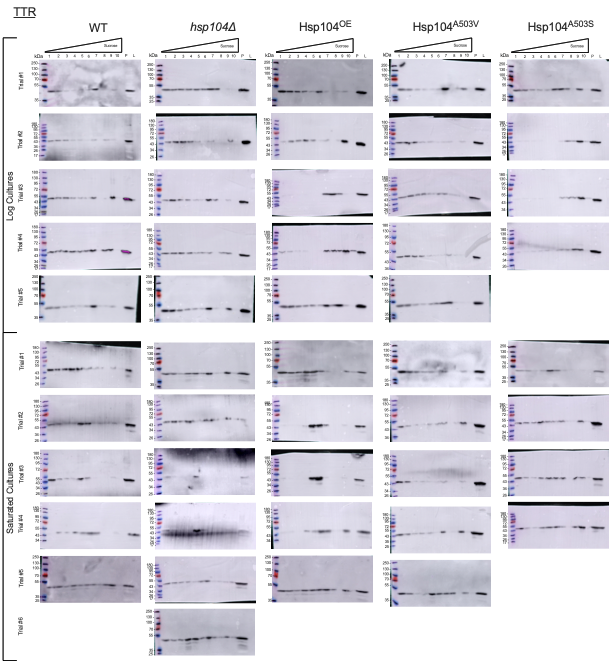
**

**Supplemental raw blots E. Raw Western blots of sucrose gradients. Relevant to Figures 3A, 4C, 5B, and Supplemental Figure 5.**


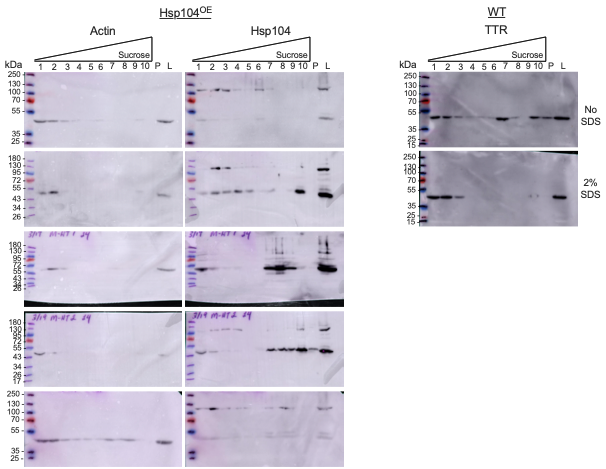


**Supplemental raw blots F. Raw Western blots of sucrose gradients. Relevant to Figures 3B and Supplemental Figure 5B.** Note that TTR antibody remains visible when detecting the Hsp104 antibody using at HRP luminol detection system. Actin antibody is detected using an AP-CDPstar detection system.


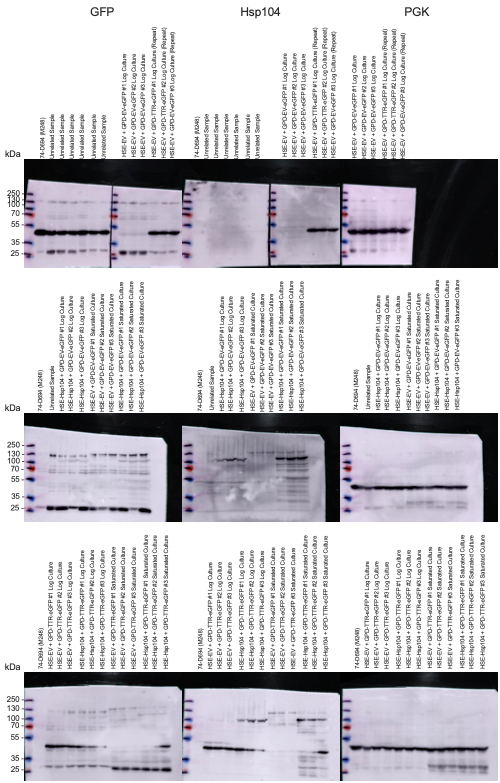


**Supplemental raw blots G. Raw Western blots. Relevant to Supplemental Figures 2B&C.** Note that TTR antibody and Hsp104 antibody is using at HRP luminol detection system and PGK is using an AP-CDPstar detection system.

Literature Cited

Chernoff, Y.O., Lindquist, S.L., Ono, B., Inge-Vechtomov, S.G., and Liebman, S.W. (1995). Role of the chaperone protein Hsp104 in propagation of the yeast prion-like factor [psi+]. Science *268*, 880-884.

Derkatch, I.L., Chernoff, Y.O., Kushnirov, V.V., Inge-Vechtomov, S.G., and Liebman, S.W. (1996). Genesis and variability of [PSI] prion factors in Saccharomyces cerevisiae. Genetics *144*, 1375-1386.

Derkatch, I.L., Uptain, S.M., Outeiro, T.F., Krishnan, R., Lindquist, S.L., and Liebman, S.W. (2004). Effects of Q/N-rich, polyQ, and non-polyQ amyloids on the de novo formation of the [PSI+] prion in yeast and aggregation of Sup35 in vitro. Proc Natl Acad Sci U S A *101*, 12934-12939.

Huh, W.K., Falvo, J.V., Gerke, L.C., Carroll, A.S., Howson, R.W., Weissman, J.S., and O'Shea, E.K. (2003). Global analysis of protein localization in budding yeast. Nature *425*, 686-691.

Jackrel, M.E., DeSantis, M.E., Martinez, B.A., Castellano, L.M., Stewart, R.M., Caldwell, K.A., Caldwell, G.A., and Shorter, J. (2014). Potentiated Hsp104 variants antagonize diverse proteotoxic misfolding events. Cell *156*, 170-182.

Malinovska, L., Kroschwald, S., Munder, M.C., Richter, D., and Alberti, S. (2012). Molecular chaperones and stress-inducible protein-sorting factors coordinate the spatiotemporal distribution of protein aggregates. Mol Biol Cell *23*, 3041-3056.
